# The authors' reply: ‘Comment on: “Effect of vitamin D monotherapy on indices of sarcopenia in community‐dwelling older adults: a systematic review and meta‐analysis” by Prokopidis et al.’

**DOI:** 10.1002/jcsm.13087

**Published:** 2022-09-09

**Authors:** Konstantinos Prokopidis, Panagiotis Giannos, Konstantinos Katsikas Triantafyllidis, Konstantinos S. Kechagias, Jakub Mesinovic, Oliver C. Witard, David Scott

**Affiliations:** ^1^ Department of Musculoskeletal Biology, Institute of Life Course and Medical Sciences University of Liverpool Liverpool UK; ^2^ Society of Meta‐research and Biomedical Innovation London UK; ^3^ Department of Life Sciences, Faculty of Natural Sciences Imperial College London London UK; ^4^ Department of Nutrition & Dietetics Musgrove Park Hospital, Taunton & Somerset NHS Foundation Trust Taunton UK; ^5^ Department of Metabolism, Digestion and Reproduction, Faculty of Medicine Imperial College London London UK; ^6^ Institute for Physical Activity and Nutrition (IPAN), School of Exercise and Nutrition Sciences Deakin University Burwood VIC Australia; ^7^ Department of Medicine, School of Clinical Sciences at Monash Health Monash University Clayton VIC Australia; ^8^ Centre for Human and Applied Physiological Sciences, Faculty of Life Sciences and Medicine King's College London London UK

In their letter to the editor, Cheng et al.^1^ performed additional meta‐regression analyses using age stratification, based on our meta‐analysis of the effects of vitamin D supplementation monotherapy on indices of sarcopenia.^2^ We did not perform age‐stratified subgroup analyses, because it is important for systematic reviews and meta‐analyses to follow *a priori* analysis plans registered in the international prospective register of systematic reviews (PROSPERO).

The authors categorized older adults into ‘young‐old’ (60–69 years old), ‘middle‐old’ (70–79 years old) and ‘old‐old’ (≥80 years old) age strata and showed that vitamin D supplementation decreased general physical performance in the middle‐old population (standardized mean difference [SMD]: −0.15; 95% CI: −0.27 to −0.02), but not young‐old or old‐old populations. The classification of such age strata is not well established,^3^ and it should be noted that significant heterogeneity exists in the health and physical function of older adults of similar chronological ages.^4^ Regardless, the meta‐regression by Cheng et al. essentially confirms our findings.

In relation to the authors' statement highlighting the need for larger scale vitamin D trials in different groups across old age, several studies,^5^, ^6^, ^7^, ^8^, ^9^, ^10^ including ours, have observed unfavourable effects on muscle strength, physical performance and risk of falls, particularly with higher doses of vitamin D. We included in our discussion that ‘… mechanistic studies may be preferable to investigate this relationship and any randomized controlled trials of high‐dose vitamin D supplementation should potentially be restricted to those at low risk of falling’. Moreover, given our own and other recent meta‐analyses that have demonstrated no effect of vitamin D on muscle strength/physical performance,^2^, ^5^ there is limited benefit to conducting further trials in the general population of older adults. Indeed, in a trial sequential analysis that determined the effects of vitamin D supplementation on falls and fractures, Bolland et al.^11^ concluded that ‘… vitamin D supplementation does not have meaningful clinical benefits …’ and that ‘Further similar trials are unlikely to alter the conclusions …’. These findings suggest that strong rationale is required to support the need for further studies on vitamin D supplementation for improving musculoskeletal health. Hence, we reiterate our main conclusion that any future trials of vitamin D supplementation for prevention or treatment of sarcopenia should be targeted at specific populations, such as those at greatest risk for low vitamin D status and its associated complications.

## Conflict of interest

There is no conflict of interest.
